# The Application of a High-Energy Fluidic Microfluidizer System Improves the Physicochemical and Antioxidant Properties of Whole Mulberry Juice

**DOI:** 10.3390/foods14132311

**Published:** 2025-06-30

**Authors:** Xuemei He, Xinyi Li, Yayuan Tang, Xiaolin Meng, Zhen Wei, Baoshen Li, Taotao Dai, Xixiang Shuai, Zhenxing Wang, Xuechun Zhang

**Affiliations:** 1 Guangxi Key Laboratory of Fruits and Vegetables Storage-Processing Technology, Guangxi Academy of Agricultural Sciences, Nanning 530007, China; xuemeihe1981@126.com (X.H.);; 2 College of Biological and Food Engineering, Southwest Forestry University, Kunming 650224, China; 3 Key Laboratory for Forest Resources Conservation and Utilization in the Southwest Mountains of China, Ministry of Education, Southwest Forestry University, Kunming 650224, China; 4 State Key Laboratory of Food Science and Technology, Nanchang University, Nanchang 330096, China; 5 South Subtropical Crop Research Institute, China Academy of Tropical Agricultural Sciences, Zhanjiang 524013, China

**Keywords:** high-energy fluidic microfluidizer, mulberry juice, stability, nutritional properties

## Abstract

Whole mulberry juice (WMBJ) was prepared using a novel high-energy fluidic microfluidizer (HEFM). The effects of varying treatment pressures (0–120 MPa) on the physical stability and nutritional quality of the juice were investigated. As the pressure increased, the average particle size (D[4,3]) decreased from 232.46 μm to 38.27 μm. This indicated that the pulp particles became smaller and more evenly dispersed, resulting in an increase in the apparent viscosity. At 90 MPa, the precipitation weight ratio increases, the turbidity value is the lowest, and the physical stability is significantly improved. Furthermore, the HEFM treatment exhibited a favorable impact on the soluble solids, pH value, total acid content, color, and antioxidant activity of the WMBJ. The contents of anthocyanins, ascorbic acid, and total polyphenols in the WMBJ reached their zenith with the 90 MPa treatment. The results demonstrated that WMBJ, characterized by its excellent physical stability and high nutritional value, can be effectively prepared through the utilization of HEFM technology. This technological approach represents a novel method for industrial WMBJ production that is both efficient and environmentally friendly while ensuring the preservation of the product’s quality.

## 1. Introduction

Mulberry (*Morus nigra* L.) is an agricultural product that has applications in both medicine and food production. This plant food resource is characterized by a high concentration of active ingredients, including polysaccharides, organic acids, amino acids, and a variety of vitamins [1]. Notably, it exhibits a substantial abundance of anthocyanins, phenolic acids, ascorbic acid, and other compounds of a similar chemical nature. The subject of this study has been shown to have remarkable antioxidant effects, and could contribute to the prevention of various diseases, including cancer, diabetes, and cardiovascular diseases [2]. However, mulberries’ perishability and their short shelf-life present significant challenges during the stacking process. The process is prone to generating high temperatures and promoting mold growth, neither of which are conducive to transportation and storage. Consequently, mulberries are frequently processed into various products, including jam, wine, and juice, among others. Additionally, they are extensively utilized in the natural dye and cosmetics industries [3]. The fruit juice processing industry has been identified as a promising solution to the challenges posed by mulberry fruit storage. Creating juice from mulberry fruit not only addresses the logistical concerns associated with their storage but also represents a significant opportunity to foster the healthy and sustainable growth of the mulberry industry [4].

A considerable quantity of nutrient-rich fruit pomace is produced during juice production, including the juicing and filtering stages. Discarding the fruit’s peel has been demonstrated to result in several undesirable consequences. Chief among these is waste generation, which is both a resource expenditure and a contributor to environmental contamination. Additionally, this practice engenders disparities between the quality of juice extracted from the fruit without peel in comparison to juice obtained from the fruit in its entirety [3]. In recent years, the global market has witnessed development opportunities due to the increasing consumer demand for juice that is free of additives, minimally processed, and highly nutritious [5]. Consequently, the production of full-component juice, which retains edible components such as pulp, has emerged as a prevailing trend. Full-component juice represents an innovative approach to juice production. By processing the entire fruit (including the peel, pulp, and seeds) to create nutrient-dense beverages, this method significantly reduces byproduct generation compared to that with conventional juice extraction techniques, while achieving the 100% utilization of raw materials.

High-pressure homogenization (HPH) technology utilizes high pressure (50–400 MPa) and physical techniques, such as cavitation, turbulence, and shear force, to process soft or semi-soft particulate materials. However, its efficacy is diminished in materials with high viscosity or those containing hard particles, and it may damage equipment [6]. Dynamic high-pressure microfluidics (DHPM) is a novel non-thermal processing technology [7]. The application of high pressure results in the generation of high shear stress, turbulence, and cavitation effects, thereby facilitating the emulsification and homogenization of materials, and also enhancing the nutritional components of the materials [8]. DHPM has been extensively employed in the production of various fruit and vegetable pulps, including cucumber and carrot juices, among others [9,10]. However, the presence of larger pulp particles has been shown to impede the flow channels of microporous materials. Consequently, its application is typically restricted to processed clarified juices that have undergone filtration, a process that often results in the depletion of nutritional components.

Novel high-energy fluidic microfluidizers (HEFM) are equipped with two-stage crushing equipment. The process begins with a preliminary crushing stage that employs wet pre-treatment, followed by the refinement of the materials using microfluidic equipment. The system’s functionality encompasses the processing of both simple and complex full-component foods [11]. The high-pressure jet mill has been demonstrated to modify the physical and chemical properties of materials, enhance their content of active substances, and alter their size [2]. A study demonstrated that, in comparison with a combination of a colloid mill and a homogenizer, whole soybean milk processed by HEFM exhibited reduced particle sizes, a more uniform oil droplet and protein distribution, a looser microstructure, and, concurrently, an increased stability and soy isoflavone content [11]. Furthermore, pea starch particles subjected to processing by HEFM undergo substantial expansion while their structural integrity is maintained. Additionally, the gelatinization viscosity and viscoelasticity of these particles are augmented [12]. In the case of peanut milk, an increase in pressure leads to the amalgamation of proteins combined with oil droplets, resulting in the formation of a stable three-dimensional network structure. This process concomitantly enhances the flavor substance content [13].

The present study had two objectives: first, to use a high-energy fluidic microfluidizer (HEFM) to prepare whole mulberry juice (WMBJ), and second, to investigate how the HEFM process affects the physical stability and nutritional value of the resulting WMBJ. Physical stability was evaluated based on parameters such as the particle size, zeta potential, precipitate weight ratio, turbidity, and rheological properties. Nutritional value was evaluated based on parameters such as color changes, nutritional components, and antioxidant activity. The results reflect the changes in the WMBJ’s quality and stability during HEFM processing, facilitating its use in juice production.

## 2. Materials and Methods

### 2.1. Materials

Fresh ripe mulberry samples were collected from a local farmer’s market in Nanning, China. Gallic acid, Folin–Ciocalteu reagents, and L-ascorbic acid were obtained from McLean Biochemical Technology Co., Ltd. in Shanghai, China. D-(+)-Galacturonic acid, a total antioxidant capacity assay kit based on the ABTS method, and the relevant reagents were purchased from Solarbio Technology Co., Ltd. (Beijing, China).

### 2.2. Preparation of Whole Mulberry Juice by HEFM

The whole mulberry juice (WMBJ) was prepared using HEFM (State Key Laboratory of Food Science and Technology, Nanchang University, Nanchang, China), which comprised a pre-pulverizer unit and a high-energy fluidic microfluidizer. The fresh mulberries were washed to remove any impurities. Subsequently, water and mulberry fruit were incorporated at a mass ratio of 1:1 (*w*/*w*), and a wet grinder was employed for preliminary crushing to yield a coarse pulp. Subsequently, the coarse pulp was subjected to treatment with high-pressure jet mill equipment at pressures of 30, 60, 90, and 120 MPa. The obtained whole mulberry juice (WMBJ) was labeled as WMBJ_M-30_, WMBJ_M-60_, WMBJ_M-90_, and WMBJ_M-120_, respectively, with the outlet temperatures being 35 °C, 46 °C, 58 °C, and 62 °C, respectively. The sample that had not been treated with the high-pressure jet mill but had been treated with the wet crushing equipment was labeled as WMBJ_M-0_. All samples were stored in a dark environment at −20 °C.

### 2.3. Effect of HEFM on the Stability of WMBJ

#### 2.3.1. Particle Size and Particle Size Distribution

The determination of the particle size and particle size distribution (PSD) of WMBJ was conducted using a laser diffraction particle size analyzer (Mastersizer 3000, Malvern Instruments Co., Ltd., Worcestershire, UK). The refractive index of the particles was set to 1.57, the particle absorption rate was set to 0.001, and water was used as the dispersant (with a refractive index of 1.33). The results of the study included key particle size parameters such as the volume–average particle size D[4,3], the surface area–average particle size D[3,2], and the cumulative distribution percentages D(10), D(50), and D(90).

#### 2.3.2. Rheological Properties

The rheological properties of WMBJ were determined using a rheometer (TA DHR-1, Waters-TA Instruments, New Castle, DE, USA), with the size of the parallel plate gap set to 1 mm. In a controlled environment maintained at a constant temperature of 25 °C, 1.5 mL of WMBJ was extracted and positioned within the designated sensing area. The shear rate was varied from 0.1 to 100 s^−1^, and a viscosity curve was plotted. The Herschel–Bulkley model (Equation (1)) was employed to model its fluidity, as this model has been widely applied to describe the rheological properties of pseudoplastic fluid materials.

(1) $$\tau =\tau_{0}+K\cdot \gamma^{n}$$

The symbol τ denotes the shear stress (Pa); τ_0_ indicates the yield stress (Pa); K represents the consistency coefficient (Pa·s), γ symbolizes the shear rate (s^−1^); and n is the flow behavior index.

#### 2.3.3. Turbidity and Precipitate Weight Ratio

The determination of turbidity was executed following the method outlined by [14], with minor modifications. Following the thorough mixing of the sample, it was subjected to a centrifugal process at an ambient temperature for 20 min. The supernatant of the sample was extracted, the absorbance of which was measured at a wavelength of 660 nm. Deionized water served as the blank control. The determination of the precipitate weight ratio was carried out according to the method provided in [15] with appropriate adjustments. The sample, weighing 20 g, was accurately weighed and placed in a centrifuge tube. The centrifuge was operated at 25 °C and 4000 revolutions per minute (rpm) for 15 min. The precipitate weight ratio was calculated according to Equation (2):

(2) $$\varphi \left(\%\right)=(M_{1}/M_{2})\times 100$$

The symbols M_1_ and M_2_ are the weight of the beverage and the weight of the precipitate, respectively.

### 2.4. Effect of HEFM on the Physicochemical and Nutritional Indicators of WMBJ

#### 2.4.1. pH, Soluble Solids, Total Acidity, and Color Attributes

The sample’s pH value was measured using a pH meter (Model PHS—3C, Qiwei Industrial Co., Ltd., Hangzhou, China) at room temperature. A portable sugar–acid analyzer (PAL-BX, Atago Co., Ltd., Tokyo, Japan) was employed to determine the content of soluble solids (TSS) and the total acidity (TA) of the sample. To determine the total acidity, the juice was subjected to centrifugation for 15 min. Subsequently, the supernatant was collected, diluted, and measured. A portable colorimeter (CS—412, Caipu Industrial Co., Ltd., Hangzhou, China) was employed to conduct a color analysis on the WMBJ sample. The lightness L* value, a* value (red–green), and b* value (yellow–blue) of the sample were recorded, and the total color difference ΔE was calculated according to Equation (3):

(3) $$∆E=\sqrt{{\left(∆L^{*}\right)}^{2}+{{\left(∆a^{*}\right)}^{2}+(∆b^{*})}^{2}}$$

#### 2.4.2. Total Anthocyanin Content

The total anthocyanin content of the WMBJ sample was determined by employing the pH differential method, using the method proposed in [16] with minor modifications. Initially, 30 mL of the acidified ethanol extractant was added to the juice. Subsequently, ultrasonic extraction was carried out at 50 °C for 10 min. Subsequent to this, the mixture underwent a centrifugation process, during which the upper layer, known as the supernatant, was meticulously collected. The resulting supernatant was then appropriately diluted. Subsequently, potassium chloride solution with a pH of 1.0 and sodium acetate buffer solution with a pH of 4.5 were added separately. The resulting solutions were then subjected to an incubation process in a water bath maintained at a temperature of 45 °C in the dark for 50 min. Subsequently, the absorbances were measured at the maximum absorption wavelengths of 700 nm and 530 nm. The anthocyanin content in the sample was calculated using Equation (4), and the results are presented in terms of the cyanidin-3-O-glucoside (C3G) equivalent (mg/100 mL).

(4) $$\mathrm{Anthocyanin}\;\mathrm{content}\;(\mathrm{mg}/100\;\mathrm{mL})=A\times MW\times DF\times V\times 100/\varepsilon \times L\times V_{1}$$

In this equation, A = (A_1_ − A_2_) − (A_3_ − A_4_), and A_1_ and A_2_ represent pH 1.0 samples measured at 530 nm and 700 nm, respectively. A_3_ and A_4_ are pH 4.5 samples measured at these same wavelengths. MW represents the molecular weight of C3G. The relative molecular mass of C3G has been determined to be 449.2 g/mol. DF is the dilution factor, V is the extraction solution volume (in mL), V1 is the sample volume (in mL), ε is the molar extinction coefficient, and L is the optical path (in cm).

#### 2.4.3. Ascorbic Acid Content

The ascorbic acid content was determined in accordance with the method proposed in [17]. The WMBJ sample was extracted with trichloroacetic acid (50 g/L), and then ultrasonicated in an ice bath for 15 min. Subsequently, the samples were subjected to a centrifugal process at a speed of 4000 rpm for 10 min. This procedure was repeated thrice. Subsequently, the collected supernatants were thoroughly mixed, and the volume was meticulously adjusted to 100 mL. The extract was then subjected to a series of chemical reactions, involving the incorporation of trichloroacetic acid (50 g/L), absolute ethanol, BP–ethanol (5 g/L), 0.4% phosphoric acid–ethanol, and FeCl_3_–ethanol (0.3 g/L). Subsequently, the specimen was placed in a water bath maintained at 30 °C in complete darkness for incubation for 60 min, referred to as the “incubation period”. Following this, the optical density of the specimen was measured at a specific wavelength of 534 nm.

#### 2.4.4. Total Polyphenol Content

As posited by [18], the Folin–Ciocalteu method was employed to ascertain the total phenolic content (TPC) of the WMBJ. Sample extraction was conducted using an 80% ethanol solution, followed by ultrasonication for 30 min. Subsequently, the sample underwent a centrifugation step for 8 min. The extract was subsequently diluted to a constant volume. The extract was then mixed with distilled water and Folin–Ciocalteu reagent. Following a 5 min incubation in the dark at room temperature, an 8% Na_2_CO_3_ solution was immediately added to the mixture. The mixture was then placed in a water bath at 30 °C and incubated in the dark for 1 h. The degree of absorption was measured at a wavelength of 760 nm, and the results were expressed as (mg/100 mL).

#### 2.4.5. Polysaccharide Content

As delineated by [19], the WMBJ samples were subjected to centrifugation to extract the supernatant. Subsequently, a fourfold amount of absolute ethanol was added and thoroughly mixed. The mixture was then subjected to a process of ethanol precipitation at a temperature of 4 °C for 4 h. Subsequent to centrifugation, the upper layer, or the supernatant, was discarded in order to obtain the extract. The total polysaccharide content was determined using the phenol–sulfuric acid method, with D-glucose serving as the standard reference material. The extract was dissolved in water and subsequently diluted to a final volume of 100 mL. The polysaccharide solution was combined with 1 mL of 5% phenol and 5 mL of concentrated sulfuric acid. The mixture had to be vigorously agitated and subsequently subjected to an incubation process in a water bath maintained at 40 °C for 30 min. Following the cooling process, the measurement of the sample’s optical density at a wavelength of 490 nm is required. The resulting values are then expressed in mg/100 mL.

#### 2.4.6. Soluble Pectin Content

The soluble pectin content was measured in accordance with the method provided in [20] with slight modifications. To commence the procedure, a precise quantity of the WMBJ sample was weighed and subsequently added to 25 mL of 95% ethanol. The mixture was subjected to a boiling water bath for 30 min, and this process was repeated multiple times until the complete removal of all other substances, including sugars. The precipitate was added to 20 mL of distilled water and maintained in a water bath at 50 °C for 30 min to facilitate the dissolution of the pectin. Following its removal and cooling to ambient temperature, the specimen was subjected to a centrifugal process at a speed of 4000 rpm for 15 min. Following centrifugation, the resultant filtrate was transferred into a 100 mL volumetric flask, and the volume was adjusted to the designated point with distilled water. Subsequently, 0.2 mL of the carbazole–ethanol solution (1.5 g/L) was added, and the solution was placed in a dark environment for 30 min. The measurement of the absorbance value was conducted at a wavelength of 530 nm, and the resultant data were expressed in mg/100 mL.

#### 2.4.7. Antioxidant Activities

The antioxidant activity was evaluated by determining the scavenging abilities of 2,2-diphenyl-1-picrylhydrazyl radical (DPPH•) and 2,2′-azino-bis(3-ethylbenzothiazoline-6-sulfonic acid) radical cation (ABTS•+). The extract was obtained following the centrifugation of the WMBJ samples. A volume of 2 mL of the extract was then mixed with 2 mL of the DPPH• solution (0.2 mm/moL), and the mixture was incubated for 30 min in the dark at room temperature. The mixture’s optical density was subsequently measured at a wavelength of 517 nm. The scavenging ability of the radical (ABTS•+) in the extract was determined according to the ABTS free radical scavenging ability test kit.

#### 2.4.8. Statistical Analysis

The determinations were replicated thrice, and the mean values and standard deviations were calculated by employing statistical analysis software (SPSS 27.0, Chicago, IL, USA). One-way analysis of variance (ANOVA) was employed to ascertain significant differences (*p* < 0.05) among the mean values of the samples.

## 3. Results and Discussion

### 3.1. Effect of HEFM on Stability of WMBJ

#### 3.1.1. Particle Size and PSD

The particle size distribution of fruit pulp exerts a notable influence on the taste profile, physical stability, and rheological properties [21]. In a suspension, the system’s stability is maximized, the sedimentation rate is decelerated, and delamination is minimized when the particle size is reduced to a minimum. Table 1 presents the particle sizes in the WMBJ processed using HEFM at different pressures. These include the volume–average particle size (D[4,3]), the surface area–average particle size (D[3,2]), and the volume distribution percentiles (D(10), D(50), D(90)). As the pressure increased, a significant decrease in the particle size was observed (*p* < 0.05). A comparison of the data revealed a marked decrease in the respective figures, with values dropping to 80.3%, 84.2%, 83.2%, 82.5%, and 84.1%, respectively, when contrasted with the WMBJM-0 baseline. It has been demonstrated that D[4,3] is more strongly influenced by large particles, while D[3,2] is highly sensitive to small particles. These findings are consistent with research on tomato juice and peanut milk [22], which demonstrated that HEFM has an exceptional effect on crushing, significantly enhancing the physical stability and sensory quality of the final products. Furthermore, the research conducted in [23,24] demonstrated that the particle sizes of the composite pear juice and frozen orange concentrated juice subjected to the DHPM treatment also exhibited a substantial decrease.

**Figure 1:**
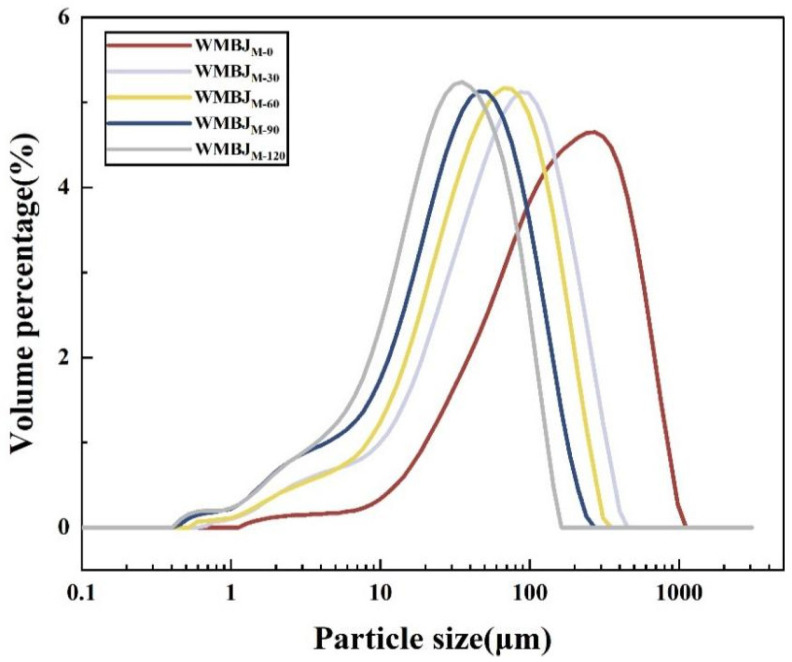
Particle size distribution of the whole mulberry juice (WMBJ) under varying HEFM treatment conditions.

As illustrated in Figure 1, the impact of HEFM on the particle size distribution (PSD) of the WMBJ samples is evident, with all of the samples exhibiting polydispersity. In the WMBJ_M-0_ sample, the maximum peak is observed at 400 μm. Conversely, for samples subjected to pressures ranging from 30 to 120 MPa, the peak positions are concentrated within the 30–100 μm range and undergo a gradual rightward shift. As the magnitude of the treatment pressure increases, the particle size distribution exhibits an upward shift, concomitantly resulting in a narrowing of its range. The proportion of small particles increases substantially, and the particle distribution becomes more homogeneous. This suggests the possibility of cell rupture, resulting in the release of cell contents and, consequently, an increase in the content yield [21]. Additionally, a novel particle size peak emerged in the 0.4–1 μm range, which is likely indicative of protein particles. As the pressure increases, the volume proportion of these particles gradually increases [25]. It has been demonstrated that the implementation of HEFM on WMBJ can effectively reduce the particle size, retard particle sedimentation, and remarkably enhance the product’s stability.

#### 3.1.2. Zeta Potential

The zeta potential is a critical metric for assessing the stability of colloidal suspensions. The absolute value of this quantity is widely employed to assess the stability of dispersed systems [26]. As demonstrated in Table 2, the zeta potential values for all of the groups are negative. Subsequent to the HEFM treatment, the cloudy juice contained a substantial number of negatively charged particles. When compared to the untreated group, no significant variation in the zeta potential was observed with changes in the treatment pressure. This phenomenon is likely attributable to the release of biopolymers such as pectin from the cell wall. The particles became enveloped by a negatively charged pectin layer, leading to an overall negatively charged surface [27]. The HEFM treatment had no marked effect on the WMBJ’s zeta potential, with its value remaining relatively stable within the range of −(5.87–8.84 mV). Accordingly, concerning the electrostatic stability, the HEFM treatment exerted negligible influence on the potential stability of the WMBJ.

#### 3.1.3. Rheological Properties

**Figure 2:**
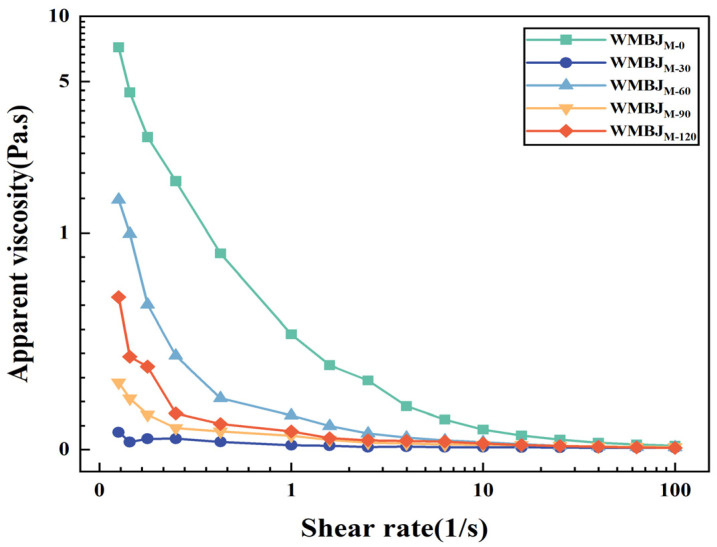
Apparent viscosity of whole mulberry juice (WMBJ) treated under various HEFM treatment conditions.

The rheological properties of foodstuffs are of pivotal significance in the evaluation of sensory quality and play a substantial role in the optimization of fruit and vegetable juice processing technologies. Achieving an appropriate viscosity can enhance the juice’s full-bodied taste [28]. As illustrated in Figure 2, the apparent viscosity of the WMBJ is shown to be contingent upon the shear rate, subsequent to the HEFM treatment. In Table 3, the calculated yield stress (τ), consistency coefficient (K), and flow index (n) for the WMBJ are presented, along with the model regression coefficients (R^2^ ranging from 0.9654 to 0.9966). As the shear rate increases, the apparent viscosity of the fluid gradually diminishes. WMBJ is a paradigmatic non-Newtonian fluid. As the homogenization pressure increases, the WMBJ experiences a gradual decrease in viscosity. Concurrently, the n value declines, while the K value rises. When the pressure reaches 120 MPa, the increase in the K value suggests an enhanced degree of shear thinning. Concurrently, the viscosity of the sample also increases, rendering the WMBJ more viscous. This phenomenon could be attributed to the fact that the HEFM treatment causes cell destruction and fragmentation, thereby increasing the surface area of the suspended particles [29]. Furthermore, an increased amount of cellular content is released into the juice, and the interaction between particles is enhanced by van der Waals forces, electrostatic forces, and hydration, resulting in an increased apparent viscosity [30]. A similar phenomenon has been validated in other studies. For instance, ref. [6] found that the apparent viscosity of orange juice concentrate first decreases and then increases. Reference [13] observed a decrease in the viscosity of tomato juice with increasing pressure. These findings suggest a potential correlation between viscosity changes and the degree of cell fragmentation and crushing effects.

#### 3.1.4. Precipitate Weight Ratio and Turbidity

**Figure 3:**
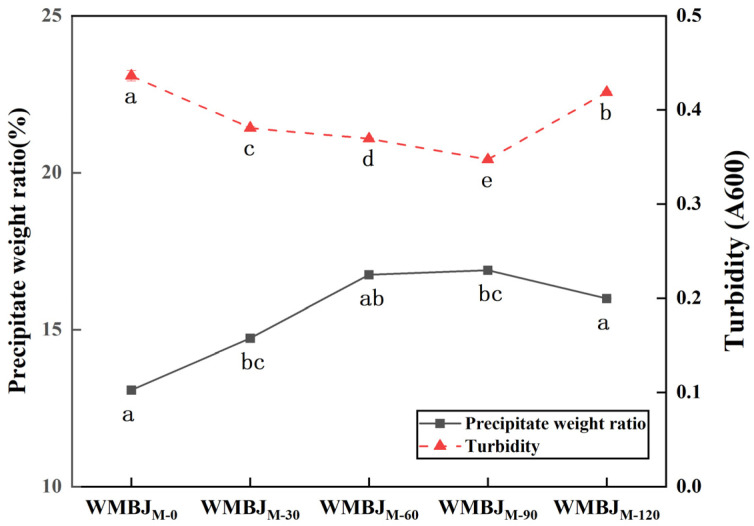
Effect of HEFM on precipitate weight ratio and turbidity of WMBJ. The different lowercase letters signify statistically significant differences in samples subjected to varying pressures (*p* < 0.05).

The precipitate weight ratio has been identified as a metric for evaluating the physical stability of fruit and vegetable juices, as well as the water-holding capacity of macromolecules [31]. As illustrated in Figure 3, the precipitate weight ratio of the WMBJ following the HEFM treatment is significantly higher than that of the untreated group (*p* < 0.05). When the pressure escalates from 0 to 90 MPa, the precipitate weight ratio climbs from 13.06% to 15.89%, peaking at 90 MPa, and then experiences a decline at 120 MPa. This finding is consistent with the results reported in the research conducted by [22] on tomato juice. Furthermore, juice that has undergone high-pressure homogenization contains a significant amount of small-size pectin and insoluble fiber, which contributes to enhancements of its water-holding capacity [32]. As the HEFM pressure increases, the turbidity value initially declines and subsequently rises. Concurrently, the comminution effect enhances the system’s water-holding capacity and suspension stability. However, it has been demonstrated that elevated levels of pressure may result in pectin depolymerization and network structure disruption [33]. The increase in pressure leads to a decrease in particles, thereby allowing greater amounts of light to penetrate the juice, and a decrease in turbidity [34]. Reference [35] also found, following high-pressure homogenization, large particles of fragmented suspended solids and small particles dispersed in the voids of large particles. This resulted in a decrease in the juice’s turbidity.

### 3.2. Effect of HEFM on Physicochemical and Nutritional Indicators of WMBJ

#### 3.2.1. pH, Soluble Solids, Total Acidity, and Color Attributes

As illustrated in Table 4, the dataset encompasses a range of pressure conditions, each leading to its own unique set of alterations in the WMBJ’s pH, total soluble solids (TSS), titratable acidity (TA), and color when treated with HEFM. The pH values of the WMBJ samples range from 3.83 to 3.89 with HEFM administration, and the pH was not statistically significantly impacted (*p* > 0.05). The soluble solids content of the WMBJ_M-0_ is 5.10 Brix°. The pressure exerted minimal influence on the process under examination; however, a marked increase in pressure is observed at 120 MPa, a finding that is likely attributable to the disruption of the cell wall by HEFM, which in turn results in the release of elevated levels of soluble solids [36]. Nevertheless, the findings in [37] demonstrated that there was no substantial alteration in the soluble solids content subsequent to the HEFM treatment of corn pulp.

The total acidity (TA) content after the HEFM treatment was found to be higher than that of the untreated group. This phenomenon may be attributed to the enhanced dissolution of organic acids under high pressure [26] and the increased ionization of weak acids, leading to an increase in H^+^ [38]. Additionally, the color parameters L*, a*, b*, and ΔE have been observed to increase gradually with increasing pressure. The increase in the L* value is attributed to the enhancement of light transmittance resulting from the reduction in particle size. The rise in the a* value is attributable to the release of pigments caused by cell destruction, while the increase in the b* value is associated with the promotion of pigment extraction at a high pressure [39]. It has been demonstrated that HEFM exerts a favorable influence on the color of mulberry juice, with no deleterious effect on its appearance.

#### 3.2.2. Total Anthocyanins

**Figure 4:**
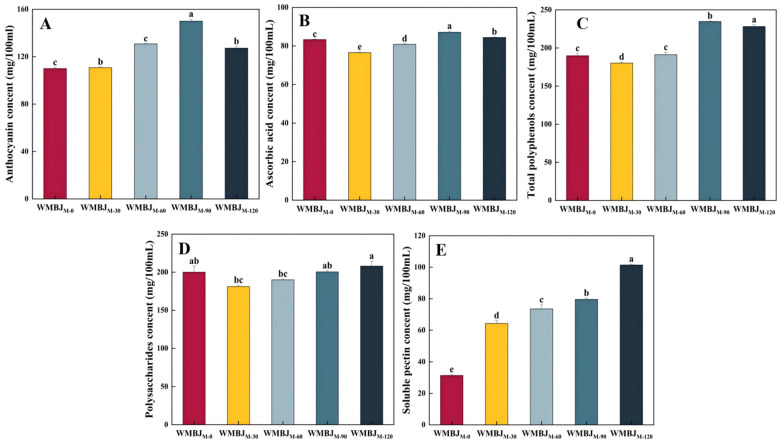
Effects of HEFM on the anthocyanin (**A**), ascorbic acid (**B**), total polyphenol (**C**), polysaccharide (**D**), and soluble pectin (**E**) content of WMBJ. The different lowercase letters signify statistically significant differences (*p* < 0.05).

Anthocyanins are flavonoid phenolic compounds that exhibit instability and are susceptible to factors such as light and heat [16]. As demonstrated in Figure 4A, with the rise in HEFM pressure, the anthocyanin content in the WMBJ initially increased and subsequently decreased. As the pressure increased from 0 to 90 MPa, there was a concomitant increase in the anthocyanin content, reaching 18.91%, 36.36%, and 15.62% of the content observed in the untreated group, respectively. This phenomenon may be attributed to the elevated degree of cell wall fragmentation, which enhances the permeability of the cell contents. This, in turn, promotes the release of anthocyanins. However, when the pressure reaches 120 MPa, there is a concomitant decrease in the anthocyanin content. This phenomenon can be attributed to the rise in temperature (up to 65 °C) induced by the increase in pressure, which expedites anthocyanin degradation. Concurrently, the extent of cell fragmentation is excessive, impeding the dissolution of active substances. These results suggest that HEFM technology can effectively promote the release of anthocyanins. However, it is important to note that excessive pressure and temperature may lead to their degradation [40]. Furthermore, ref. [41] discovered that continuous flow high-pressure homogenization not only augmented the release of anthocyanins from blueberries but also effectively stabilized the nutritional components during storage.

#### 3.2.3. Ascorbic Acid Content

Ascorbic acid demonstrates suboptimal stability, with a propensity to undergo oxidation and degradation under conditions such as elevated temperatures, pressures, and exposure to light during processing [5]. As demonstrated in Figure 4B, the ascorbic acid content in the WMBJ treated with different HEFM pressures changed significantly (*p* < 0.05). The ascorbic acid content in the WMBJ_M-0_ sample was initially measured at 83.32 mg/100 mL. Subsequent to the implementation of the HEFM treatment, a 4.6% and 1.3% increase in content was observed, respectively. It has been demonstrated that the strong cavitation effect and collisions that occur during the HEFM treatment process result in an improvement in the extraction rate of ascorbic acid in the suspended particles.

At a range of lower pressures, from 0 to 90 MPa, the release amount of ascorbic acid exceeded its destruction amount, and the content exhibited a gradual increase. However, at 120 MPa, the elevated pressure and temperature may have induced alterations in the ascorbic acid’s molecular structure, leading to a reduction in its content. Concurrently, in [17], it was discovered that an increased homogenization pressure concomitantly led to a gradual increase in the ascorbic acid content of mango juice. As demonstrated by [42], high-pressure homogenization at 150 MPa led to a substantial reduction in the ascorbic acid content in pomegranate juice. These findings suggest that the observed variations may be attributable to disparities in the sample characteristics and homogenization conditions.

#### 3.2.4. Total Polyphenols

Mulberry fruits have been found to contain a variety of polyphenolic substances, including anthocyanins, flavonoid compounds, hydrolyzable tannins, and phenolic acids. As illustrated in Figure 4C, the TPC of the WMBJ underwent a significant change following the HEFM treatment (*p* < 0.05). The WMBJ_M-0_ sample exhibited a TPC of 189.83 mg/100 mL. It demonstrated a range of pressures between 30 and 60 MPa, and no statistically significant differences (*p* > 0.05) were observed when compared to the untreated group. However, at 90 MPa, it exhibited a 23.7% increase, which disrupted the cell wall structure and reduced the particle size due to high-pressure homogenization.

Concurrently, the shear and cavitation effects induced the liberation of the polyphenols entrapped within the intricate matrix, thereby enhancing the extraction rate [43]. Conversely, an escalation in pressure to 120 MPa resulted in a decline in the TPC. This phenomenon may be attributed to the rise in temperature resulting from intensified homogenization, in addition to the prolonged exposure of the sample to oxygen, which facilitated polyphenol oxidation [44]. A similar phenomenon was observed in the study of apple juice after high-pressure treatment [45] and in the study of a mixture of carrot, apple, and peach juice [46]. Furthermore, research has demonstrated that microfluidic treatment can augment the release of polyphenols within the fiber matrix [47].

#### 3.2.5. Polysaccharides

The quantity of polysaccharides present in the WMBJ subjected to HEFM is demonstrated in Figure 4D. As the pressure increases, the dissolution number of polysaccharides initially decreases and subsequently increases. The lowest recorded value of 30 MPa (181.11 mg/100 mL) may be attributable to the dissolution of additional substances during the extraction process. This phenomenon has been observed to affect the dissolution and alcohol precipitation process of polysaccharides [48]. At a pressure of 120 MPa, the maximum polysaccharide yield is attained, reaching a value of 208.071 mg/100 mL. The underlying reason for this phenomenon may be attributed to the fact that when the WMBJ is subjected to treatment by HEFM, it is exposed to a series of forces, including a high shear force, impact force, and cavitation force. These forces, when acting in unison, result in a reduction in the particle size of the material, an increase in the contact area between the material and the dissolving substances, and a promotion of polysaccharide dissolution. This, in turn, leads to an enhancement in the polysaccharide yield [49].

#### 3.2.6. Soluble Pectin

The alterations in the soluble pectin content in the WMBJ before and after the HEFM treatment are illustrated in Figure 4E. The soluble pectin content in WMBJ_M-0_ was 30.82 mg/100 mL. Following the implementation of the HEFM treatment, a substantial increase in the soluble pectin content was observed (*p* < 0.05), with levels reaching between 1.04 and 2.03 times those of the untreated group. Additionally, an upward trend was noted in the soluble pectin levels in response to increasing pressure. This phenomenon may be attributed to the heightened degree of fragmentation and dissociation of cell tissues, which, in turn, promoted an increase in the degree of soluble pectin dissolution. Concurrently, the transformation of insoluble pectin may also result in the augmentation of its content [50]. The authors of [51] ascertained that high-pressure homogenization treatment at 20–180 MPa could substantially elevate the soluble pectin content in carrot juice. They found that the strong cavitation and shearing effects of high-pressure homogenization damaged the cell wall structure, leading to the release of pectin into the system and an increase in its content [52]. Furthermore, an increase in soluble pectin during the process of high-pressure homogenization has been demonstrated to enhance the viscosity and stability of fruit juices [53]. In [28], analogous results were obtained in the study of mango juice. The application of high-pressure homogenization resulted in an augmentation of the soluble pectin content in the mango juice. This phenomenon facilitated the coating of insoluble particles, thereby establishing a gel network that enhanced the physical stability of the sample.

### 3.3. Antioxidant Activities

**Figure 5:**
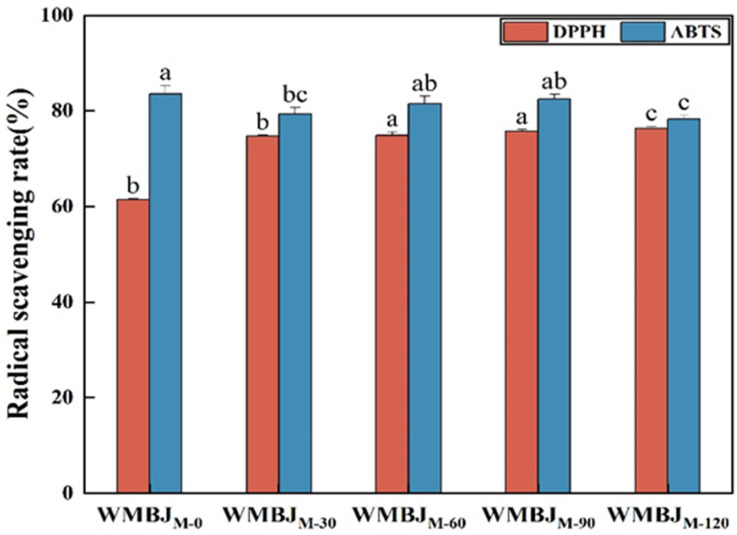
Effect of HEFM on radical scavenging activity of WMBJ. The different lowercase letters represent significant differences in samples subjected to varying pressures (*p* < 0.05).

Mulberry fruits have been found to contain high concentrations of polysaccharides and phenolic compounds, which contribute to their notable antioxidant activity. DPPH• is a stable nitrogen-centered organic radical that can be scavenged by antioxidant substances [54]. As illustrated in Figure 5, the WMBJ demonstrates a notable capacity to scavenge DPPH• under various stress conditions. Following treatment with HEFM, the scavenging rate exhibited a higher value than that of the untreated group. When exposed to 120 MPa, the scavenging ability exhibited the greatest strength, reaching 76.35%. The ABTS method was employed to assess the total antioxidant capacity. The blue-green ABTS+ radical, which is generated by oxidation, reacts with the antioxidant. The more pronounced the fading of the solution, the stronger the antioxidant capacity [55]. Following the HEFM treatment, the WMBJ’s antioxidant properties decreased, with the most significant decrease occurring at 120 MPa, resulting in a 5.3% reduction compared to the untreated group. The scavenging rates at 90 MPa and 0 MPa are 83.57% and 82.45%, respectively, with no significant difference. The literature indicates that the application of microfluidization treatment can facilitate the release of polyphenols within the fiber matrix, thereby augmenting the TPC [47]. The polyphenolic content reported in this study also exhibited an upward trend. In addition, it has been determined that during the processing of full-fat soy milk and mango juice using HEFM, there is an increase in the isoflavone and ascorbic acid content, thereby enhancing the antioxidant properties [11,17].

## 4. Conclusions

The present study investigated the effects of the HEFM treatment under varying pressures (0–120 MPa) on the physical stability and nutritional components of the WMBJ. The results demonstrated that the HEFM treatment could effectively disrupt the cell tissue structure, significantly reduce the particle size and improve its uniformity, while increasing the apparent viscosity. In the context of the treatment condition of 90 MPa, the WMBJ demonstrated optimal physical stability, as evidenced by its notably low turbidity levels and optimal sedimentation stability. Concurrently, during the treatment process, it promoted the release of cell contents and significantly increased the L* value while producing marginal increases in the a* and b* parameters, improving the product color, and enabling the anthocyanin, ascorbic acid, and total phenol contents to reach peak values. The antioxidant activity was significantly enhanced, and polysaccharide dissolution was also promoted. It is noteworthy that the treatment process exerted no substantial influence on the fundamental indicators, including the pH value, soluble solids, and titratable acidity. In summary, the HEFM treatment at 90 MPa has been demonstrated to enhance WMBJ’s physical stability and nutritional quality. This technology demonstrates considerable promise in the domain of juice processing. The implementation of this process has the potential to enhance product stability and optimize the utilization rate of raw materials.

## Figures and Tables

**Table 1:** The particle size of the whole mulberry juice treated with HEFM.

Samples	D[3,2] (μm)	D[4,3] (μm)	D(10) (μm)	D(50) (μm)	D(90) (μm)
WMBJ_M-0_	57.33 ± 3.21 ^a^	232.46 ± 4.09 ^a^	31.75 ± 3.21 ^a^	168.09 ± 1.17 ^a^	535.19 ± 12.42 ^a^
WMBJ_M-30_	22.24 ± 0.65 ^b^	91.31 ± 1.06 ^b^	10.95 ± 3.21 ^b^	68.83 ± 0.96 ^b^	205.79 ± 1.95 ^b^
WMBJ_M-60_	19.64 ± 0.63 ^b^	71.63 ± 2.45 ^c^	10.25 ± 3.21 ^b^	54.54 ± 1.76 ^c^	159.22 ± 5.54 ^c^
WMBJ_M-90_	12.68 ± 0.33 ^c^	50.48 ± 1.02 ^d^	5.62 ± 3.21 ^c^	37.98 ± 0.78 ^d^	113.38 ± 2.35 ^d^
WMBJ_M-120_	11.27 ± 0.05 ^c^	38.27 ± 0.16 ^e^	5.33 ± 3.21 ^c^	29.48 ± 0.08 ^e^	84.91 ± 0.37 ^e^

The reported results correspond to the mean ± standard deviation. Different lowercase superscript letters within the same line signify statistically significant differences (*p* < 0.05).

**Table 2:** Effects of varying HEFM treatment conditions on the zeta potential of whole mulberry juice (WMBJ).

Samples	Zeta Potential
WMBJ_M-0_	−7.27 ± 0.227 ^a^
WMBJ_M-30_	−6.25 ± 0.089 ^cd^
WMBJ_M-60_	−8.84 ± 0.070 ^bc^
WMBJ_M-90_	−5.87 ± 0.596 ^d^
WMBJ_M-120_	−7.34 ± 0.56 ^a^

The reported results correspond to the mean ± standard deviation. Different lowercase superscript letters within the same line signify statistically significant differences (*p* < 0.05).

**Table 3:** Values for the parameters of the Herschel–Bulkley model of WMBJ prepared by different HEFM treatment conditions.

Samples	τ (Pa)	K	n	R^2^
WMBJ_M-0_	0.8436 ± 0.117 ^a^	0.1194 ± 0.088 ^a^	0.6043 ± 0.126 ^ab^	0.9966
WMBJ_M-30_	0.0358 ± 0.019 ^cd^	0.0788 ± 0.089 ^a^	0.8197 ± 0.101 ^a^	0.9962
WMBJ_M-60_	0.1708 ± 0.063 ^bc^	0.0229 ± 0.006 ^a^	0.7509 ± 0.737 ^ab^	0.9911
WMBJ_M-90_	0.0221 ± 0.002 ^d^	0.0246 ± 0.009 ^a^	0.7686 ± 0.085 ^ab^	0.9984
WMBJ_M-120_	0.2505 ± 0.027 ^a^	0.0805 ± 0.017 ^a^	0.5697 ± 0.074 ^b^	0.9654

The reported results are consistent with the mean standard deviation. The presence of variation is indicated by the lowercase superscript letters on the same line, with statistical significance defined as (*p* < 0.05).

**Table 4:** Effects of different treatment conditions of HEFM on the pH, total soluble solids, total acidity, and color attributes of WMBJ.

Samples	WMBJ_M-0_	WMBJ_M-30_	WMBJ_M-60_	WMBJ_M-90_	WMBJ_M-120_
pH	3.89 ± 0.01 ^a^	3.86 ± 0.01 ^a^	3.84 ± 0.01 ^a^	3.83 ± 0.01 ^a^	3.84 ± 0.01 ^a^
TSS (Brix°)	5.10 ± 0.00 ^c^	5.10 ± 0.00 ^c^	5.13 ± 0.05 ^b^	5.20 ± 0.00 ^b^	6.03 ± 0.05 ^a^
TA (%)	0.46 ± 0.02 ^c^	0.47 ± 0.01 ^c^	0.52 ± 0.01 ^b^	0.54 ± 0.01 ^ab^	0.56 ± 0.01 ^a^
L*	24.51 ± 0.20 ^d^	25.21 ± 0.02 ^d^	25.61 ± 0.01 ^b^	25.82 ± 0.06 ^bc^	25.92 ± 0.02 ^a^
a*	0.31 ± 0.07 ^a^	0.32 ± 0.02 ^a^	0.33 ± 0.07 ^a^	0.34 ± 0.04 ^a^	0.37 ± 0.15 ^b^
b*	−3.56 ± 0.26 ^d^	−3.19 ± 0.06 ^c^	−2.71 ± 0.01 ^b^	−2.51 ± 0.13 ^b^	−1.83 ± 0.08 ^a^
ΔE	-	1.22 ± 0.04 ^d^	1.82 ± 0.02 ^c^	2.11 ± 0.13 ^b^	2.78 ± 0.02 ^a^

The reported results are consistent with the mean standard deviation. The presence of variation is indicated by the presence of lowercase superscript letters in different locations on the same line, with statistical significance defined as (*p* < 0.05).

## Data Availability

The original contributions presented in the study are included in the article, further inquiries can be directed to the corresponding author.
